# Enhancing case definitions for surveillance of human monkeypox in the Democratic Republic of Congo

**DOI:** 10.1371/journal.pntd.0005857

**Published:** 2017-09-11

**Authors:** Lynda Osadebe, Christine M. Hughes, Robert Shongo Lushima, Joelle Kabamba, Beatrice Nguete, Jean Malekani, Elisabeth Pukuta, Stomy Karhemere, Jean-Jacques Muyembe Tamfum, Emile Wemakoy Okitolonda, Mary G. Reynolds, Andrea M. McCollum

**Affiliations:** 1 Epidemic Intelligence Service, Scientific Education and Professional Development Program Office, U.S. Centers for Disease Control and Prevention, Atlanta, Georgia, United States of America; 2 Poxvirus and Rabies Branch, Division of High-Consequence Pathogens and Pathology, National Center for Emerging and Zoonotic Infectious Diseases, U.S. Centers for Disease Control and Prevention, Atlanta, Georgia, United States of America; 3 Ministry of Health, Kinshasa, Democratic Republic of Congo; 4 U.S. Centers for Disease Control and Prevention, Kinshasa, Democratic Republic of Congo; 5 Kinshasa School of Public Health, Kinshasa, Democratic Republic of Congo; 6 University of Kinshasa, Kinshasa, Democratic Republic of Congo; 7 Institut National de Recherche Biomédicale, Kinshasa, Democratic Republic of Congo; Armed Forces Health Surveillance Center, UNITED STATES

## Abstract

**Background:**

Human monkeypox (MPX) occurs at appreciable rates in the Democratic Republic of Congo (DRC). Infection with varicella zoster virus (VZV) has a similar presentation to that of MPX, and in areas where MPX is endemic these two illnesses are commonly mistaken. This study evaluated the diagnostic utility of two surveillance case definitions for MPX and specific clinical characteristics associated with laboratory-confirmed MPX cases.

**Methodology/Principal findings:**

Data from a cohort of suspect MPX cases (identified by surveillance over the course of a 42 month period during 2009–2014) from DRC were used; real-time PCR diagnostic test results were used to establish MPX and VZV diagnoses. A total of 333 laboratory-confirmed MPX cases, 383 laboratory-confirmed VZV cases, and 36 cases that were determined to not be either MPX or VZV were included in the analyses. Significant (p<0.05) differences between laboratory-confirmed MPX and VZV cases were noted for several signs/symptoms including key rash characteristics. Both surveillance case definitions had high sensitivity and low specificities for individuals that had suspected MPX virus infections. Using 12 signs/symptoms with high sensitivity and/or specificity values, a receiver operator characteristic analysis showed that models for MPX cases that had the presence of ‘fever before rash’ plus at least 7 or 8 of the 12 signs/symptoms demonstrated a more balanced performance between sensitivity and specificity.

**Conclusions:**

Laboratory-confirmed MPX and VZV cases presented with many of the same signs and symptoms, and the analysis here emphasized the utility of including 12 specific signs/symptoms when investigating MPX cases. In order to document and detect endemic human MPX cases, a surveillance case definition with more specificity is needed for accurate case detection. In the absence of a more specific case definition, continued emphasis on confirmatory laboratory-based diagnostics is warranted.

## Author summary

Human monkeypox is the most significant *Orthopoxvirus* infection since the eradication of smallpox. The disease is endemic in Africa and the majority of cases occur in the Congo Basin. Correct identification of patients is critical to deployment of efficient control measures to prevent further transmission and appropriate care for the patient. An evaluation of two surveillance case definitions revealed that the definitions had high sensitivities but low specificities for correct case identification, which results in the identification of patients with other rash illnesses. Several signs and symptoms of laboratory-confirmed monkeypox cases were identified that could be used to aid in the development of surveillance case definitions to correctly identify cases. Laboratory diagnostics continue to be an important part of correct patient identification in order to control the disease and provide adequate care.

## Introduction

Since the global eradication of smallpox, the most important *Orthopoxvirus* infection in humans in terms of ongoing numbers of cases, morbidity, and mortality has been human monkeypox (MPX) [1, 2]. Monkeypox virus (MPXV) is maintained by an enzootic cycle, with zoonotic introductions to humans often being followed by more limited human-to-human transmission [3, 4]. The animal reservoir for MPXV remains unknown, but the virus has been isolated in the wild from a squirrel (*Funisciurus anerythrus*) and a sooty mangabey (*Cercocebus atys*) [5, 6].

Infection with MPXV can lead to a smallpox-like illness characterized by a febrile prodrome, lasting 1–4 days, followed by a slowly progressing rash. The rash proceeds from macules to papules to vesicles to pustules to crusts and finally to desquamation. This occurs over a period of two to four weeks. At any given site on the body, the rash is generally in the same stage of development (e.g., all vesicles), and the lesions are typically circumscribed, umbilicated, deep-seated and firm. The rash has a centrifugal distribution, with a concentration of lesions on the extremities and face. As with smallpox, MPX lesions often appear on the palms of the hands and soles of the feet. Lymphadenopathy (inguinal, axillary, and/or cervical) is common in MPX patients and can occur prior to or at the onset of rash. Ocular infection with MPXV can lead to permanent corneal scarring and blindness [7–9].

Infection with varicella zoster virus (VZV) has a similar presentation to that of MPXV infection, and in areas where MPXV is endemic these two illnesses are commonly mistaken [10, 11]. However, there are several features of illness that typically set one infection apart from the other. For example, VZV patients typically exhibit a short, mild period of febrile prodrome, or none at all, followed by a quickly evolving (1–2 day) pleomorphic rash (i.e., a rash for which neighboring lesions may be in different stages of development). VZV lesions also often have irregular borders, and are superficial on the surface of the skin (relative to those of MPX). In addition, varicella lesions often appear in a centripetal distribution on the body [9, 12]. Although noted in rare occurrences, lesions on the palms of the hands and soles of the feet are not hallmarks of VZV infections [13]. VZV patients do not typically have pronounced lymphadenopathy, and, thus, the presence of lymphadenopathy is one distinguishing characteristic that can differentiate MPX from both smallpox and varicella. Additional illnesses that can be mistaken for MPX are other herpetic infections (in addition to VZV), drug eruptions, syphilis, yaws, scabies, and rickettsialpox [9]. Specimen collection followed by laboratory testing can be difficult to accomplish for all suspected cases in MPX endemic areas. A clinical case definition capable of enhancing the distinction between MPX and other illnesses would be useful to enable more accurate and expedient case detection, collection of higher-quality surveillance data, and improved patient management.

MPXV is enzootic in western and central Africa, with the overwhelming majority of human infections reported each year from the forested areas of the Congo Basin of Democratic Republic of Congo (DRC) [2]. MPX is a nationally reportable disease in DRC and has been identified as one of the country’s priority diseases of epidemic potential. On a bi-weekly basis, notifications of suspected MPX cases from each of the country’s Health Zones are submitted to national public health authorities; few of the suspected cases are formally investigated (i.e., case investigation forms completed and diagnostic specimens collected).

This study evaluates the diagnostic utility of two surveillance case definitions for MPX. Both definitions were herein applied to a cohort of suspected MPX cases that were identified over the course of a 42 month period via surveillance in one Province in DRC. This cohort is unique in that the dataset contained an in-depth list of signs/symptoms. The accuracy of case classification was determined using laboratory findings. We assessed clinical features of illness in patients with confirmed MPXV infection to identify characteristics distinctly associated with disease presentation and suggest modifications to the MPX surveillance case definition to improve specificity.

## Methods

### Suspect case detection and laboratory classification

Data from suspect MPX cases were obtained by investigation in accordance with national guidelines. Patients were identified as suspect MPX cases if they had a vesicular or pustular eruption with deep-seated, firm pustules and at least one of the following symptoms: fever preceding the eruption, lymphadenopathy (inguinal, axillary, or cervical), and/or pustules or crusts on the palms of the hands or soles of the feet. For suspect cases, a MPX-specific case investigation form was completed and, in most instances, two or more diagnostic specimens were collected from each suspected MPX case. The specimens were sent to the Institut National de Recherche Biomédicale (INRB) in Kinshasa for diagnostic testing. One specimen from each individual was tested at INRB for the presence of *Orthopoxvirus* DNA signatures using a real-time PCR assay [14]. If the initial PCR was negative for *Orthopoxvirus*, a second real-time PCR assay specific for VZV-specific DNA signatures was conducted (reagents provided by the United States Army Medical Research Institute of Infectious Diseases). DNA extracted at INRB and additional independent specimens, if available, were shipped to the Poxvirus Laboratory at the U.S. Centers for Disease Control and Prevention (CDC). At the CDC, DNA was extracted from original specimens and all specimens were tested with MPXV and VZV-specific real-time PCR assays [15, 16].

An individual was classified as a laboratory-confirmed MPX case if at least one specimen was 1) positive with the *Orthopoxvirus*-specific assay, and/or 2) positive by MPX-specific real-time PCR. An individual was independently classified as a laboratory-confirmed VZV case if a crust specimen tested positive for VZV DNA signatures at INRB or if an original vesicular swab or crust specimen tested positive for VZV at CDC.

### Ethical statement

These activities were determined to not be research by a CDC human subjects advisor.

### Dataset and variable inclusion for analyses

**Fig 1:**
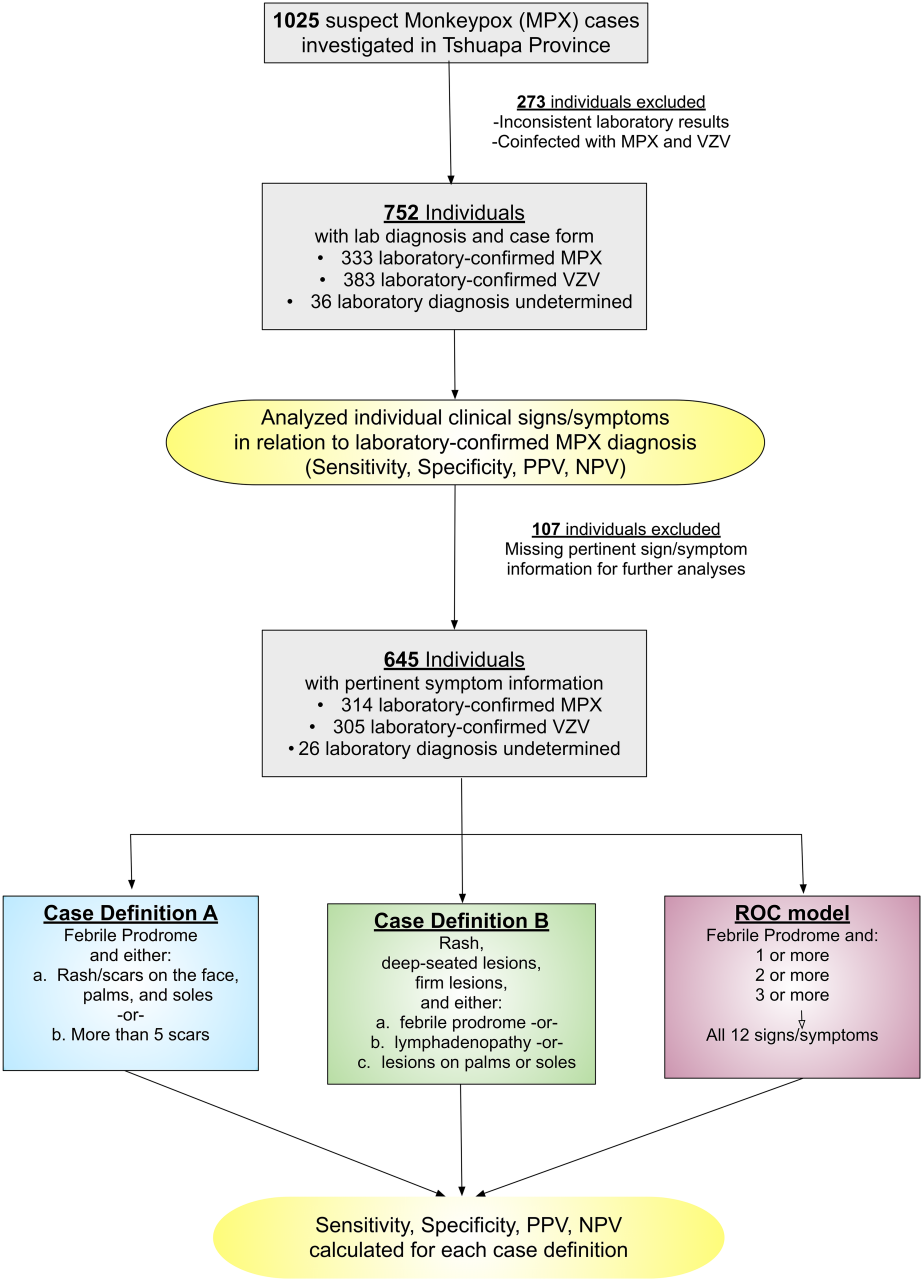
Flow chart of the cases selected for each analysis.

Suspect MPX cases in Tshuapa Province, DRC, with rash onset occurring between September 2009 and February 2014 were included in the analysis (N = 1025, Fig 1). These individuals were all assessed by a surveillance officer, who determined the individual met the surveillance case definition for a suspect MPX case. Individuals whose laboratory test results were suggestive of a coinfection with both MPXV and VZV, and those with incomplete or inconsistent laboratory results were excluded from analyses (n = 273). A total of 752 cases were included for further analyses; 333 laboratory-confirmed MPX cases, 383 laboratory-confirmed VZV cases, and 36 cases that were determined to not be either MPX or VZV (laboratory diagnosis undetermined).

Clinical signs and symptoms were recorded as checkboxes—‘yes’, ‘no’, and ‘do not know’—on the case investigation form. If an individual case investigation form had a response (‘yes or ‘no’) for any signs/symptoms, but the absence of a response for another specific sign/symptom the variable was coded as “missing” for the specific sign/symptom without a response. Febrile prodrome status was determined by either a) selection of ‘yes/no’ for the febrile prodrome variable on the case investigation form, or, if that information was missing, by, b) presence of prodrome was ascertained by determining the time interval between onset of fever and that of rash, when the information was available. The presence of lymphadenopathy was determined using the individual lymphadenopathy-type fields (inguinal, axillary, cervical). If at least one category of lymphadenopathy was reported as present, then the individual was coded “yes” for lymphadenopathy; if all fields were “no”, the individual was coded “no” for lymphadenopathy. If one or more of the fields were missing and others were “no”, the individual was coded “missing” for lymphadenopathy.

Two surveillance case definitions were evaluated in this study. According to Case Definition A, a suspect case is an individual with fever followed by a vesicular or pustular rash with the following symptoms: rash on palms, soles, and face; or the presence of 5 variola-like scars. Case Definition A has been recommended for use in endemic areas.

Case Definition B was developed as a discriminatory case definition with the inclusion of several criteria that distinguish MPX from VZV. Case Definition B encompasses individuals who have a vesicular or pustular eruption with deep-seated, firm pustules and at least one of the following symptoms: fever preceding the eruption, lymphadenopathy (inguinal, axillary, or cervical), and/or pustules or crusts on the palms of the hands or soles of the feet.

To evaluate the diagnostic accuracy of each case definition, the dataset was restricted to individuals who had all the information needed for classification using *both* case definitions (i.e., one dataset was used for the independent analyses of both definitions). A total of 645 of the 752 cases (85.8%) had sufficient information to be included in the analyses of both case definitions; 314 laboratory-confirmed MPX cases, 305 laboratory-confirmed VZV cases, and 26 cases that were determined to be neither MPX nor VZV (Fig 1). Individuals met the criteria for Case Definition A if they had a febrile prodrome and either the presence of a) rash/scars on the face, palms, and soles, or b) more than 5 scars. Individuals met the criteria for Case Definition B if they had rash with deep-seated, firm lesions and either a) febrile prodrome, b) lymphadenopathy, or c) lesions on the palms of the hands or soles of the feet. This dataset was also used for the receiver operating characteristic analysis.

### Statistical analysis

The frequencies of each sign/symptom were calculated for all cases included in the dataset, and individually for laboratory-confirmed MPX and VZV cases. Associations between reported signs/symptoms and laboratory determined diagnoses were calculated using chi-squared and Fisher exact tests. Signs/symptoms that occurred with significantly different frequency (p<0.05) between laboratory-confirmed MPX and VZV cases were further assessed for their individual sensitivity, specificity, PPV and NPV in relation to the confirmed diagnosis of MPX.

For the analysis of the two case definitions, real-time PCR diagnostic test results (for MPX and VZV) were used as the ‘gold standard’ to establish MPX and VZV diagnoses. The sensitivity, specificity, positive predictive values (PPV), and negative predictive values (NPV) were computed for the two case definitions.

A receiver operating characteristic (ROC) analysis was completed using ‘fever before rash’ and various summed frequencies of the 12 signs/symptoms that were identified as having high (>80%) sensitivities or specificities for laboratory-confirmed MPX cases. For example, individuals with ‘fever plus rash’ and one of these 12 signs/symptoms were categorized as having ‘1 criteria’; individuals with ‘fever plus rash’ and 12 signs/symptoms were ranked as ‘12 criteria’. Each of these 12 signs/symptoms counted equally to the sum. For example, if an individual reported two symptoms of ‘nausea’ and ‘cough’, they were categorized the same as an individual who reported two symptoms of ‘fatigue’ and ‘conjunctivitis’. ‘Fever before rash’ was identified as a mandatory sign/symptom because this has been consistently noted in the literature for MPX patients and also was observed at a frequency of 98.1% for all suspect cases and 99.1% for laboratory-confirmed MPX patients in the present analysis. The sensitivity, specificity, PPV and NPV for each model (with increasing number of signs and symptoms from 1 to 12) were assessed.

All data analysis was performed using SAS version 9.3.

## Results

Characteristics of the Population: A total of 752 suspect cases were included in the analysis. Approximately 53% of suspect cases were male and 46% were female; this proportion remained consistent after laboratory case classification. The mean age of suspect cases was 17 years (median 13, range 0.01–86). Of these suspect cases, 333 (44.3%) individuals were classified as laboratory-confirmed MPX cases and 419 (55.7%) were classified as laboratory-confirmed VZV (383) or undiagnosed (36) cases (Table 1).

**Table 1:** Characteristics of suspect monkeypox (MPX) cases identified between December 2009 and February 2014 (n = 752).

	Total	Laboratory-confirmed MPX cases	Laboratory-confirmed VZV	Undiagnosed cases^a^
All suspect cases	752 (100%)	333 (44.3%)	383 (50.9%)	36 (4.8%)
Sex^b^ Male Female Missing	397 (52.9%) 349 (46.4%) 6 (0.8%)	178 (53.4%) 154 (46.2%) 1 (0.3%)	205 (53.5%) 174 (45.4%) 4 (1.0%)	14 (38.9%) 21 (58.3%) 1 (2.8%)
Age (years) Mean Median Range Missing	17.04 13.33 0.01–86 3	5.77 13.82 0.08–67 n/a	18.15 13 0.01–86 2	16.98 14.72 0.8–46 1

^a^ Undiagnosed cases are those that were determined to not be either MPX or VZV.

^b^ Percentages for sex are within column percentages

Performance of specific clinical characteristics: With the objective of improving the specificity and PPV of the case definitions under examination, associations between the reported clinical signs/symptoms and a laboratory-confirmed diagnosis of MPX (versus VZV) were investigated. Significant (p<0.05) differences between laboratory-confirmed MPX and VZV cases were noted for the signs/symptoms of nausea, cough, lymphadenopathy (overall and each site), mouth ulcers, sore throat, malaise, fatigue, conjunctivitis, sensitivity to light, and bedridden (Table 2). Rash characteristics that were significantly different included same size, deep-seated, firm lesions, and the presence of lesions on the arms, legs, palms of the hands, soles of the feet, and genitals. Each of the significant signs/symptoms and rash characteristics occurred more frequently in laboratory-confirmed MPX cases than in laboratory-confirmed VZV cases. The majority of significant signs/symptoms (15/20) occurred in more than 50% in laboratory-confirmed MPX cases.

**Table 2:** Association between clinical characteristics and laboratory-confirmed monkeypox (MPX) or varicella (VZV) case classification.

Sign or Symptom	All suspect cases (752)			Laboratory-confirmed MPX cases (333)			Laboratory-confirmed VZV cases (383)			p-value^a^
	n^b^	N^b^	%	n	N	%	n	N	%	
Fever	732	737	99.3%	329	329	100%	371	376	98.7%	0.0644
Rash	718	750	95.7%	316	332	95.2%	367	382	96.1%	0.5594
Febrile Prodrome	727	741	98.1%	327	330	99.1%	371	379	97.9%	0.1965
Nausea	140	737	19.0%	75	328	22.9%	58	374	15.5%	**0.0131**
Cough	347	744	46.6%	192	331	58.0%	135	378	35.7%	**<0.0001**
Lymphadenopathy	562	731	76.9%	277	325	85.2%	265	371	71.4%	**<0.0001**
Axillary	369	737	50.1%	191	327	58.4%	166	375	44.3%	**0.0002**
Cervical	416	736	56.5%	206	326	63.2%	195	375	52.0%	**0.0028**
Inguinal	323	738	43.8%	168	326	51.5%	142	377	37.7%	**0.0002**
Chills	593	744	79.7%	262	328	79.9%	302	381	79.3%	0.8400
Mouth ulcers	333	741	44.9%	190	326	58.3%	131	381	34.4%	**<0.0001**
Sore Throat	451	738	61.1%	246	325	75.7%	188	378	49.7%	**<0.0001**
Headache	548	729	75.2%	243	322	75.5%	283	373	75.9%	0.9011
Pruritis	379	734	51.6%	170	321	53.0%	196	378	51.9%	0.7701
Malaise	453	720	62.9%	228	319	71.5%	211	367	57.5%	**0.0001**
Fatigue	590	740	79.7%	278	328	84.8%	286	377	75.9%	**0.0032**
Conjunctivitis	126	739	17.1%	79	328	24.1%	43	377	11.4%	**<0.0001**
Sensitivity to light	198	729	27.2%	105	323	32.5%	86	372	23.1%	**0.0057**
Bedridden	137	743	18.4%	95	327	29.1%	41	381	10.8%	**<0.0001**
Rash characteristics										
Monomorphic	573	658	87.1%	286	319	89.7%	264	312	84.6%	0.0585
Same size	581	657	88.4%	291	319	91.2%	264	311	84.9%	**0.0063**
Deep-seated and firm	621	660	94.1%	310	319	97.2%	286	314	91.1%	**0.0011**
Rash site										
Face	736	743	99.1%	330	333	99.1%	372	375	99.2%	0.8838
Thorax	723	743	97.3%	328	333	98.5%	362	375	96.5%	0.0973
Arms	704	742	94.9%	326	332	98.2%	345	375	92.0%	**0.0002**
Legs	331	352	94.0%	191	197	97.0%	125	138	90.6%	**0.013**
Palms	677	742	91.2%	324	333	97.3%	319	374	85.3%	**<0.0001**
Soles	604	743	81.3%	309	333	92.8%	264	375	70.4%	**<0.0001**
Genitals	144	692	20.8%	87	309	28.2%	52	349	14.9%	**<0.0001**

^a^ Chi-square test between laboratory-confirmed MPX and VZV cases. Significant p-values (p<0.05) are in bold.

^b^ n is the number of cases reported to have the sign/symptom and N is the total number of cases with data present.

Variables with high sensitivity for MPX (≥80%) were lymphadenopathy, fatigue, and the following rash characteristics: same size, deep-seated firm lesions, presence on the arms, legs, palms of the hands, and soles of the feet (Table 3). Nausea, conjunctivitis, bedridden, and lesions present on the genitals were signs/symptoms with a high specificity (≥80%), but none of these, individually, were found in laboratory-confirmed MPX cases at a frequency > 32%.

**Table 3:** Diagnostic measures^a^ of significant^b^ clinical signs/symptoms.

Symptom	Sensitivity^c^	Specificity^c^	PPV	NPV
Nausea	22.87% (18.32–27.41)	**84.11% (80.56–87.65)**	53.57% (45.31–61.83)	57.62% (53.66–61.59)
Cough	58.01% (52.69–63.32)	62.47% (57.80–67.14)	55.33% (50.1–60.56)	64.99% (60.30–69.68)
Lymphadenopathy	**85.23% (81.37–89.09)**	29.80% (25.35–34.25)	49.29% (45.15–53.42)	71.60% (64.80–78.40)
Axillary	58.41% (53.07–63.75)	56.59% (51.79–61.38)	51.76% (46.66–56.86)	63.04% (58.11–67.98)
Cervical	63.19% (57.95–68.43)	48.78% (43.94–53.62)	49.52% (44.71–54.32)	62.50% (57.20–67.80)
Inguinal	51.53% (46.11–56.96)	62.38% (57.70–67.06)	52.01% (46.56–57.46)	61.93% (57.26–66.60)
Mouth ulcers	58.28% (52.93–63.63)	65.54% (60.97–70.11)	57.06% (51.74–62.37)	66.67% (62.09–71.24)
Sore Throat	75.69% (71.03–80.36)	50.36% (45.54–55.19)	54.55% (49.95–59.14)	72.47% (67.31–77.64)
Malaise	71.47% (66.52–76.43)	43.89% (39.03–48.75)	50.33% (45.73–54.94)	65.92% (60.23–71.60)
Fatigue	**84.76% (80.87–88.65)**	24.27% (20.13–28.41)	47.12% (43.09–51.15)	66.67% (59.12–74.21)
Conjunctivitis	24.09% (19.46–28.71)	**88.56% (85.49–91.64)**	62.70% (54.25–71.14)	59.38% (55.49–63.27)
Sensitivity to light	32.51% (27.4–37.62)	77.09% (73.01–81.18)	53.03% (46.08–59.98)	58.95% (54.76–63.13)
Bedridden	29.05% (24.13–33.97)	**89.90% (87.01–92.8)**	69.34% (61.62–77.06)	61.72% (57.85–65.59)
Rash characteristics				
Same size	**91.85% (88.85–94.85)**	14.79% (11.01–18.58)	50.43% (46.36–54.50)	65.79% (55.12–76.46)
Deep-seated and firm	**97.18% (95.36–99.00)**	8.80% (5.79–11.80)	49.92% (45.99–53.85)	76.92% (63.70–90.15)
Rash site				
Arms	**98.19% (96.76–99.63)**	7.80% (5.21–10.40)	46.31% (42.62–49.99)	**84.21% (72.62–95.80)**
Legs	**96.95% (94.55–99.35)**	9.68% (5.02–14.33)	57.70% (52.38–63.03)	71.43% (52.11–90.75)
Palms	**97.30% (95.56–99.04)**	13.69% (10.36–17.02)	47.86% (44.10–51.62)	**86.15% (77.76–94.55)**
Soles	**92.79% (90.02–95.57)**	28.05% (23.70–32.40)	51.16% (47.17–55.15)	**82.73% (76.45–89.02)**
Genitals	28.16% (23.14–33.17)	**84.86% (81.27–88.45)**	60.00% (52.03–67.97)	59.41% (55.30–63.53)

^a^ Each diagnostic measure is presented with the 95% confidence interval.

^b^ Significant clinical characteristics were chosen from a comparison of laboratory-confirmed MPX and VZV cases, Table 2.

^c^ Diagnostic measures with values ≥80% are in bold.

Analysis of the case definitions: Two-by-two tables and diagnostic values for the two case definitions are presented in Tables 4 and 5. Two hundred ninety-one (92.6%) laboratory-confirmed MPX cases satisfied Case Definition A; and 306 (97.5%) laboratory-confirmed MPX cases satisfied Case Definition B; 245 (74%) and 303 (91.5%) non-MPX cases [laboratory-confirmed VZV cases and undiagnosed (MPX and VZV negative) cases] satisfied Case Definitions A and B, respectively.

**Table 4:** The number of suspect monkeypox (MPX) cases that were captured by Case Definitions A and B by their MPX laboratory case classification.

Laboratory- confirmed MPX Case ^a^	Case Definition A			Case Definition B		
	Captured	Not captured	Total	Captured	Not captured	Total
Yes	291	23	314	306	8	314
No	245	86	331	303	28	331
Total	536	109	645	609	36	645

^a^ MPX case classification determined by laboratory test results.

**Table 5:** Summary of the diagnostic parameters for both case definitions.

Parameter	Case Definition A^a^	Case Definition B^a^
Sensitivity	92.68% (89.79–95.56)	97.45% (95.71–99.20)
Specificity	25.98% (21.26–30.71)	8.46% (5.46–11.46)
PPV	54.29% (50.07–58.51)	50.25% (46.28–54.22)
NPV	78.90% (71.24–86.56)	77.78% (64.20–91.36)

^a^ % (95% confidence interval)

The sensitivity of both case definitions was high, with the value for the Case Definition B a bit higher than that of Case Definition A (97.45% vs. 92.86%, respectively). Similarly, the specificity of both case definitions was low and the Case Definition A had a higher specificity (25.98%) than Case Definition B (8.46%). The PPVs were similar for both definitions (50.25% for Case Definition B and 54.92% for Case Definition A), as were the NPVs (77.78% for Case Definition B and 78.90% for Case Definition A). The NPV was higher than the PPV for both definitions.

**Fig 2:**
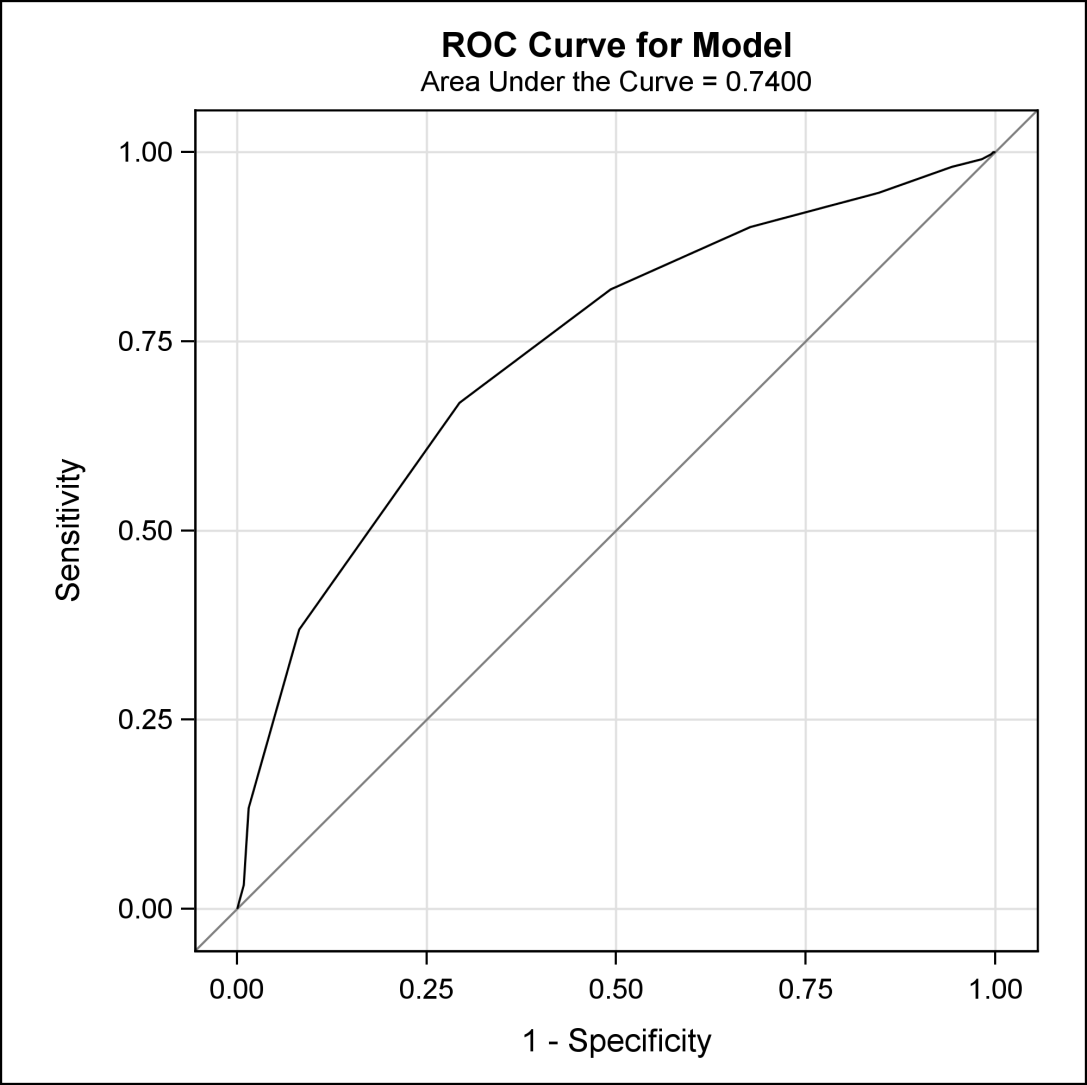
A receiver operating characteristic analysis showing the area under the curve for the model using the symptom ‘fever before rash’ and various summed frequencies of 12 signs/symptoms.

Receiver operating characteristic analysis: Using the 12 identified signs/symptoms with high sensitivity and/or specificity values, the ROC analysis tested the performance and accuracy of 12 models (with increasing numbers of signs and symptoms) (Table 6). In general, models with a greater number of signs/symptoms (>8 but <12) demonstrated excellent specificity (>90%) but low sensitivity (<40%). In contrast, models with a lower number of signs/symptoms (<7) had excellent sensitivity (>90%) but low specificity (<40%). The models for MPX cases that had the presence of ‘fever before rash’ plus at least 7 or 8 of the 12 signs/symptoms demonstrated a more balanced performance between sensitivity and specificity. There was greatly improved specificity for the models that included 7 (50.76%) or 8 (70.69%) signs/symptoms when compared to either Case Definition A (25.98%) or B (8.46%). The area under the curve for the model using these summed symptom counts was 0.74 (Fig 2).

**Table 6:** Accuracy of the minimum number of signs/symptoms in predicting laboratory-confirmed monkeypox cases.

No. signs/ symptoms^a^	1	2	3	4	5	6	7	8	9	10	11	12
**Sensitivity**	100%	99.68%	99.04%	98.09%	94.59%	90.13%	81.85%	66.88%	36.94%	13.38%	3.18%	0%
**Specificity**	0.30%	0.60%	1.81%	5.74%	15.41%	32.33%	50.76%	70.69%	91.84%	98.49%	99.09%	100%
**PPV**	48.76%	48.75%	48.90%	49.68%	51.47%	55.82%	61.19%	68.40%	81.12%	89.36%	76.92%	---
**NPV**	51.24%	51.25%	51.10%	50.32%	48.53%	44.18%	38.81%	31.60%	18.88%	10.64%	23.08%	---

^a^ Sum of the minimum number of highly sensitive or highly specific signs/symptoms present for a suspect monkeypox case that also had ‘fever before rash’

## Discussion

The choice and utility of a case definition will be guided by its intended use. Case Definition A was designed to detect a single case of MPX illness, followed by an immediate outbreak response and control efforts. Case Definition B was designed to be a discriminatory definition used in the context of surveillance for disease, to better understand the extent and burden of disease in an endemic area. Both case definitions were characterized by high sensitivities but very low specificities. The high values for sensitivity were expected since the dataset represented patients who were diagnosed with suspected MPX virus infection (prior to laboratory confirmation). These characteristics indicate that both definitions are useful for screening purposes and are well-designed for outbreak detection. Given that MPX is an endemic, regularly occurring, reportable disease in Tshuapa Province, the case definitions should be sufficient to capture true MPX cases, such that local or national officials may want to launch an outbreak response if they observe an aberration or threshold in the number or rate of reported infections. For surveillance purposes, however, especially in resource-limited countries such as DRC, it is necessary for a definition to capture all true cases and at the same time minimize the number of false positives. Attributes of Case Definition B, including a low specificity and moderate PPV, are not optimal for the objectives of disease surveillance. Observations of a moderate PPV and high NPV for both case definitions is consistent with a disease with a low prevalence in the population. Although prominent and regularly occurring, MPX does have a relatively low incidence in Tshuapa Province with a cumulative incidence rate of 4.8/10,000 over a four year period (data available upon request).

Several signs/symptoms had a high sensitivity (lymphadenopathy, fatigue, and the following rash characteristics: same size, deep-seated firm lesions, presence on the arms, legs, palms of the hands, and soles of the feet), which indicates that these signs/symptoms may be useful in ruling out other febrile rash illnesses that may be circulating in an area of active MPXV transmission. Signs/symptoms with a high specificity (nausea, conjunctivitis, bedridden, lesions present on the genitals), on the other hand, may be useful in identifying true MPX cases. These four signs/symptoms and, also, sensitivity to light were characteristics that occurred more frequently in laboratory-confirmed MPX cases than VZV cases. However, none of these discriminatory signs/symptoms were found at a high frequency in MPX cases (<50%). Thus, these signs/symptoms may be eligible components of a case definition to identify MPX cases, however, they cannot be a mandatory component.

Case definitions for MPX include characteristics of disease presentation specific to the rash itself. This analysis reinforced the inclusion of lesion size and surface presentation (deep-seated vs superficial). Notably, the “characteristic” lesion locations of palms of the hands and soles of the feet, although present in higher frequency in MPX cases than VZV cases, were prevalent in all suspect cases (91.2% and 81.3%, respectively) and were not helpful in increasing the specificity for MPX cases. Lesions on the palms and soles have been previously noted in VZV cases in central Africa [13], and the findings here indicate that this presentation may be more common than recognized before.

The benefit of an ROC analysis is that one is able to evaluate the effect of increasing number of signs and symptoms on the sensitivity and specificity of case identification. Both laboratory-confirmed MPX and VZV cases presented with many of the same signs and symptoms. Instead of limiting the case definition to an all-or-nothing analysis, we chose to limit the ROC analysis to a subset of highly sensitive and specific signs/symptoms. As such, the analysis indicated that the combination of 7 or 8 signs/symptoms was the most optimal model to accurately predict laboratory-confirmed MPX cases. This analysis emphasized the utility of including the minimum 12 signs/symptoms (nausea, conjunctivitis, bedridden, lesions on genitals, lymphadenopathy, fatigue, lesions of the same size, deep-seated firm lesions, lesions present on the arms, legs, palms of the hands, or soles of the feet) on a MPX-specific case investigation, followed by the classification of a patient as a suspect MPX case if they possessed a combination of any 7 or 8 of these specific signs/symptoms plus ‘fever before rash’. Suspected MPX patients are rarely followed over the course of their infection. Patients are often only seen once, the data on clinical signs/symptoms is assessed and captured at that single time point, and no further follow-up is conducted. A definition such as the one suggested by the ROC analysis may allow for greater flexibility and utility in detecting true MPX cases at any given point during the course of the infection, since it allows for the presence of 7 or 8 signs/symptoms (versus 12). Additional evaluations to discern a specific suite of signs/symptoms that can be easily identified by healthcare personnel in endemic areas are warranted. This could be followed by modification of the surveillance investigation tool to incorporate the 12 signs/symptoms and evaluation of the utility of a new, modified case definition that accounts for the presence of 7 or 8 signs/symptoms when determining if a patient is a suspected MPX case.

The population used in this analysis was a population of suspect MPX cases identified through surveillance for human MPX illness in DRC. A total of 137 cases were excluded from the final analysis due to incomplete data from case report forms (missing signs/symptoms). The majority of excluded cases were laboratory-confirmed VZV cases (78 or 72.9% of those excluded), which may lead to a slight bias in the dataset that contains relatively more laboratory-confirmed MPX cases than were identified in the surveillance dataset. However, a similar proportion of laboratory-confirmed MPX and VZV cases were used for the analyses. Further, data from the suspect MPX cases was collected at one point in time during their illness. The dataset is unique in that it captured many signs/symptoms present for patient; similar datasets with a large number of patients/cases are not available for independent comparison. This data does not represent the spectrum of signs/symptoms that a patient may experience over the course of their illness, and, the result may be a limitation in the frequency of signs/symptoms for suspect cases. Nevertheless, this data reflected the range of presentations of signs/symptoms recognized in suspected MPX patients in an area with endemic disease.

In order to document and detect endemic human MPX cases, a surveillance case definition with more specificity in accurate case detection is needed. In rural DRC there are increasingly limited resources, competing health priorities, and a lack of regional testing capacity, which emphasizes the need to easily and efficiently deploy a case definition to accurately identify true MPX patients and limit false positives. A single MPX case or the decision to launch an outbreak response requires considerable resources. According to national guidelines, once a MPX case is identified, the case should be isolated and contacts should be followed for 21 days. Strict recommendations regarding hygiene and infection control are instituted for the entire period of illness, which can last for four weeks. In the absence of a more specific case definition, continued emphasis on laboratory-based diagnostics is warranted. More rapid and efficient methods of diagnosing suspect MPX patients, via a regional surveillance laboratory or a clinical laboratory, are needed to better identify and care for patients followed by appropriate control measures.

**Disclaimer**: The findings and conclusions in this report are those of the author(s) and do not necessarily represent the views of the Centers for Disease Control and Prevention.
